# Fine Mapping of a Pleiotropic Locus (*BnUD1*) Responsible for the Up-Curling Leaves and Downward-Pointing Siliques in *Brassica napus*

**DOI:** 10.3390/ijms24043069

**Published:** 2023-02-04

**Authors:** Mao Yang, Jun Chen, Yuqing Chang, Shubei Wan, Zisu Zhao, Fei Ni, Rongzhan Guan

**Affiliations:** State Key Laboratory for Crop Genetics and Germplasm Enhancement, Nanjing Agricultural University, Nanjing 210095, China

**Keywords:** *Brassica napus*, downward-pointing silique, gene mapping, up-curling leaf

## Abstract

Leaves and siliques are important organs associated with dry matter biosynthesis and vegetable oil accumulation in plants. We identified and characterized a novel locus controlling leaf and silique development using the *Brassica napus* mutant *Bnud1*, which has downward-pointing siliques and up-curling leaves. The inheritance analysis showed that the up-curling leaf and downward-pointing silique traits are controlled by one dominant locus (*BnUD1*) in populations derived from NJAU5773 and Zhongshuang 11. The *BnUD1* locus was initially mapped to a 3.99 Mb interval on the A05 chromosome with a BC_6_F_2_ population by a bulked segregant analysis-sequencing approach. To more precisely map *BnUD1*, 103 InDel primer pairs uniformly covering the mapping interval and the BC_5_F_3_ and BC_6_F_2_ populations consisting of 1042 individuals were used to narrow the mapping interval to a 54.84 kb region. The mapping interval included 11 annotated genes. The bioinformatic analysis and gene sequencing data suggested that *BnaA05G0157900ZS* and *BnaA05G0158100ZS* may be responsible for the mutant traits. Protein sequence analyses showed that the mutations in the candidate gene *BnaA05G0157900ZS* altered the encoded PME in the trans-membrane region (G45A), the PMEI domain (G122S), and the pectinesterase domain (G394D). In addition, a 573 bp insertion was detected in the pectinesterase domain of the *BnaA05G0157900ZS* gene in the *Bnud1* mutant. Other primary experiments indicated that the locus responsible for the downward-pointing siliques and up-curling leaves negatively affected the plant height and 1000-seed weight, but it significantly increased the seeds per silique and positively affected photosynthetic efficiency to some extent. Furthermore, plants carrying the *BnUD1* locus were compact, implying they may be useful for increasing *B. napus* planting density. The findings of this study provide an important foundation for future research on the genetic mechanism regulating the dicotyledonous plant growth status, and the *Bnud1* plants can be used directly in breeding.

## 1. Introduction

Leaf morphology affects dicotyledonous plant photosynthetic activities and the accumulation of dry matter. The slight up-curling of leaves may increase the overall light energy use efficiency and improve plant yields. Leaf shape formation is a complex developmental process involving leaf primordium formation, polarity establishment, and cell differentiation. The curled leaf phenotype is caused by mutations to genes related to leaf development [1,2], including genes encoding the transcription factors Class III HOMEODOMAIN LEUCINE-ZIPPER (HD-Zip III) [3,4,5], KANADI (KAN) [6,7,8], WUSCHEL RELATED HOMEOBOX (WOX) [9], and TB1-CYC-PCF (TCP) [10]. These transcription factors modulate leaf polarity establishment and cause leaves to curl upward by regulating the expression of leaf asymmetry development-related genes, including *ASYMMETRIC LEAVES 1* (*AS1*) and *AS2* [11]. Plant hormone biosynthesis and signal transduction is another major factor influencing leaf shapes. For example, auxin response factors (e.g., ARF3) can induce leaf curling by affecting the mutual antagonism between HD-ZIP III and KAN transcription factors [12]. The *UCU1* gene, which encodes the SHAGGY/GSK3 protein, is involved in auxin and Brassinosteroid (BR) signal transduction. A mutation to this gene can lead to the formation of leaves that curl downward as well as dwarfism [13]. Both miRNA165 and miRNA166 affect leaf development by regulating *HD-ZIP III* gene expression [4,14,15], whereas miRNA160 causes leaves to curl by regulating the expression of the auxin-responsive genes *ARF10* and *ARF17* [16,17,18]. In addition, a few non-coding RNAs reportedly contribute to the establishment of adaxial-abaxial polarity, which may lead to the development of curled leaves [19]. The leaf curling-related genes have also been associated with cutin and cuticular wax production and cell wall synthesis.

Siliques are specific to plants in the family Brassicaceae, which consists of numerous important species, including *Arabidopsis thaliana*, *Brassica napus*, and *Brassica rapa*. Siliques are organs that store seeds, which contain oil and protein. Silique traits, such as length, diameter, angle (upright, downward pointing, or oblique), seed number, stalk, and fruit shaft, directly affect the seed yield [20,21]. Accordingly, the genes controlling silique growth and development must be identified and functionally annotated. Previous studies on silique traits focused on the seed number per silique, silique length, silique number per plant, 1000-seed weight of siliques, and silique dehiscence resistance [22,23,24,25,26]. However, the genetic basis for the silique angle remains unknown. An earlier study on the *A. thaliana brevipedicellus* mutant revealed that a mutation to the *AtBP* gene encoding a homeodomain protein (KNAT1) is responsible for the formation of a downward-pointing pedicel and flower [27]. The *AtBP* gene is expressed in the peripheral zone of the shoot apical meristem and in the cortical cell layers of the inflorescence stem (peduncle) and pedicel [28]. A mutation to the *BP* gene alters its asymmetrical expression patterns in the pedicel abaxial and adaxial sides, thereby severely affecting pedicel cell differentiation, elongation, and growth, leading to downward-pointing pedicels [27].

Cell and organ shapes in plants are dependent on the chemical structure and mechanical properties of the extracellular matrix. The pectin matrix, which is the main load-bearing plant cell wall component, is constantly re-modeled to enable plant morphological development. This re-modeling is regulated by several loosening and stiffening agents, including pectin methylesterase (PME) and calcium ions. Specifically, PMEs are ubiquitous enzymes that can catalyze the demethylesterification of homogalacturonan (HG) [29]. The demethylesterified HG can either form Ca^2+^ bonds, which promote the formation of the so-called ‘egg-box’ model structure that solidifies the cell wall, or it serves as the substrate of other pectin-degrading enzymes that loosen the cell wall [30,31]. The continuous and precise spatiotemporal regulation of the activities of these agents is necessary for proper morphogenesis at the cell and tissue levels [32,33].

The down-regulated expression of *PME* genes may decrease the extent of the pectin demethylation in the cell wall and restrict the binding between Ca^2+^ and PMEs, leading to severe decreases in the cell wall texture and rigidity, which may substantially alter the plant organ phenotype. However, relatively few *PME* genes have been functionally characterized. In *A. thaliana*, a PME may also contribute to the downward-pointing flower and silique phenotype [34,35]. The overexpression of pectin methylesterase inhibitor (PMEI)-encoding genes and the down-regulated expression of *PME* genes in wild-type *A. thaliana* result in plants with curled leaves, a convoluted shoot, and misshapen siliques [34].

Oilseed rape (*Brassica napus* L.) is one of the most important oil crops worldwide because it is a source of high-quality edible oil that is consumed by humans, while also serving as protein-rich feed for animals and a raw material for industrial processes. As the global population continues to increase, there is a growing demand for higher yielding oilseed rape cultivars. The leaf and silique morphological traits can directly affect photosynthesis and rapeseed yields. Hence, studies on rapeseed mutants with abnormal leaf and silique traits may be relevant for the breeding of dicotyledonous plants with enhanced characteristics and future research on silique developmental biology.

Leaf morphological mutations in *B. napus* (e.g., upward and downward curling and wrinkling) are related to photosynthetic efficiency and affect the yield. Previous research on leaf curling revealed the associated loci and genes, including a dominant locus (*BnDWF/DCL1*) [36], semi-dominant genes (*sca* and *ds-4*) [37,38], and the genes (*BnUC1*, *BnUC2*, and *BnUC3*) [39,40,41] responsible for the up-curling of *B. napus* leaves. Analyses of gene functions demonstrated that abnormalities in some leaf flatness-related traits are caused by the deficient expression of genes encoding proteins mediating phytohormone (e.g., auxin and brassinolide) biosynthesis or signaling [13,42,43,44]. Defects in genes directly affecting phytohormone regulatory pathways usually result in severe morphological changes that lead to abnormal plant development, with potential implications for breeding. However, identifying loci associated with leaf morphological variations may help developmental biologists and breeders attempting to develop new varieties with enhanced photosynthetic activities.

In the present study, we analyzed a *B. napus* cv. NJAU5773 mutant (*Bnud1*) that had downward-pointing siliques and up-curled leaves using a genetic and molecular approach. We fine mapped a dominant locus (*BnUD1*), identified candidate genes for the mutated pleiotropic traits on the basis of sequencing and gene expression experiments, and explored the effects of *BnUD1* on agronomic traits. Our findings may serve as the foundation for the breeding of *B. napus* lines with potentially improved morphological characteristics and may be useful for elucidating the genetic mechanism underlying leaf and silique development.

## 2. Results

### 2.1. Performance of the Bnud1 Mutant

**Figure 1:**
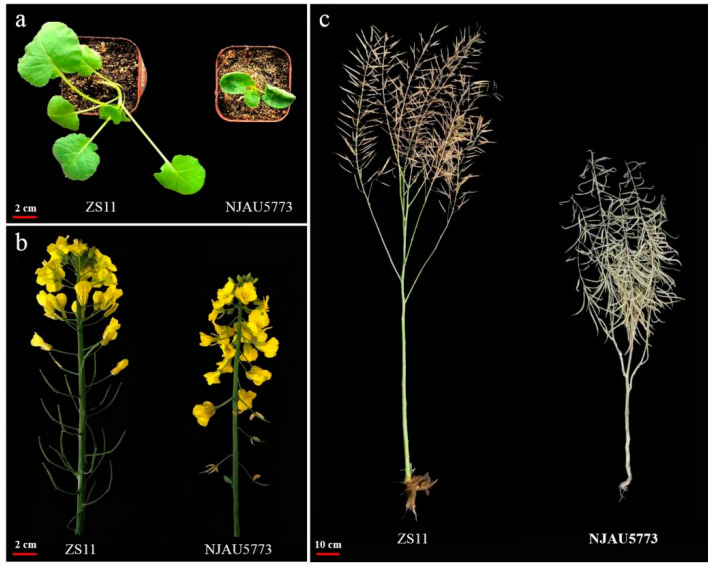
Performance of the parents NJAU5773 and ZS11 for constructing mapping population. (**a**) shows the leaves of the parent NJAU5773 with up-curling leaves (**right**) and the parent ZS11 with flat leaves (**left**) at the seeding stage. (**b**) shows the flowers of the parent NJAU5773 with downward-pointing flowers (**right**) and the parent ZS11 with normal flowers (**left)** at the flower stage. (**c**) shows the siliques of the parent NJAU5773 with downward-pointing silique (**right**) and the parent ZS11 with upright silique (**left**) at the maturity stage.

*Brassica napus* NJAU5773 (named *Bnud1* in this study) has up-curling leaves at the seedling stage, which is in contrast to the normal flat leaves of the canola variety Zhongshuang 11 (ZS11) (Figure 1a). Additionally, NJAU5773 has downward-pointing flowers that develop into downward-pointing siliques (Figure 1b,c). Plants with up-curling leaves and downward-pointing siliques exhibit semi-dwarfism in a segregating population derived from NJAU5773. To examine the *Bnud1* mutant, near isogenic lines (ZS11-UD1 and ZS11) were developed via marker-assisted selection.

### 2.2. Inheritance of the Up-Curling Leaf and Downward-Pointing Silique Traits

In the current study, NJAU5773 was reciprocally crossed with canola variety ZS11, which has a sequenced genome (http://brassicadb.org/brad/, accessed on 15 January 2023), to generate F_1_ plants and progeny populations for genetic analyses. Investigations involving segregating populations, which included F_2_, and successively backcrossed populations indicated that the segregation ratio (i.e., plants with up-curling leaves and downward-pointing siliques vs. wild-type plants) was consistent with the Mendel inheritance ratio (as suggested by the Chi-square test) (Table 1). Accordingly, the up-curling leaf and downward-pointing silique traits appear to be controlled by a dominant locus (i.e., *BnUD1*).

### 2.3. Mapping of the BnUD1 Locus

To map the *BnUD1* locus, a BC_6_F_2_ population consisting of 308 plants was derived from the cross between the two parents (NJAU5773 and ZS11). Thirty plants with up-curling leaves and downward-pointing siliques and 30 plants with flat leaves and upright siliques were selected from the BC_6_F_2_ population to scan the *BnUD1* locus via a bulked segregant analysis (BSA) approach. The two pooled samples were sequenced, with 30.06× sequence coverage for plants with the *BnUD1* locus and 21.65× sequence coverage for plants lacking the *BnUD1* locus. The clean reads were aligned to the *B. napus* cv. ZS11 reference genome (ZS11-v20200127; http://cbi.hzau.edu.cn/cgi-bin/rape/download_ext, accessed on 15 January 2023). This helped to identify the single nucleotide polymorphisms (SNPs) between the two pools. A total of 444,519 SNPs and 79,892 small insertion/deletions (InDels) were detected (Table S1). The ΔSNP index was calculated and the following two segments of the A05 chromosome were considered as candidate *BnUD1*-harboring regions: 7,094,487–11,086,188 bp (approximately 3.99 Mb) and 37,261,266–37,670,607 bp (approximately 0.41 Mb) (Figure S1; Table S2).

**Figure 2:**
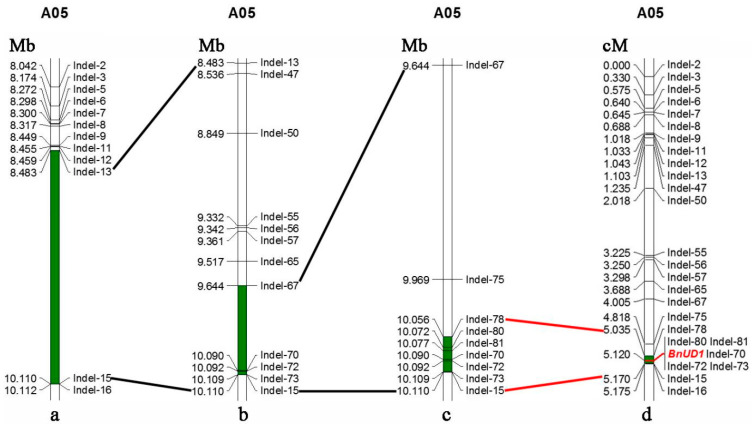
Mapping the leaf up-curling and silique downward-pointing locus *BnUD1*. (**a**) indicates that the *BnUD1* locus was preliminary mapped in an interval of 1.63 Mb (shaded in green) between InDel markers InDel-13 and Indel-15 (black lines) on A05 chromosome. (**b**) shows that the *BnUD1* locus was blue-colored in the physical interval of 466.58 kb (shaded in green) with facilitation of the developed InDel markers. (**c**) shows that the *BnUD1* locus was fine mapped in the physical interval of 54.84 kb (shaded in green). (**d)** shows that *BnUD1* was in a 0.135 cM region between InDel markers Indel-78 and Indel-15 (red lines), shaded in green. The *BnUD1* locus is highlighted in red.

**Figure 3:**
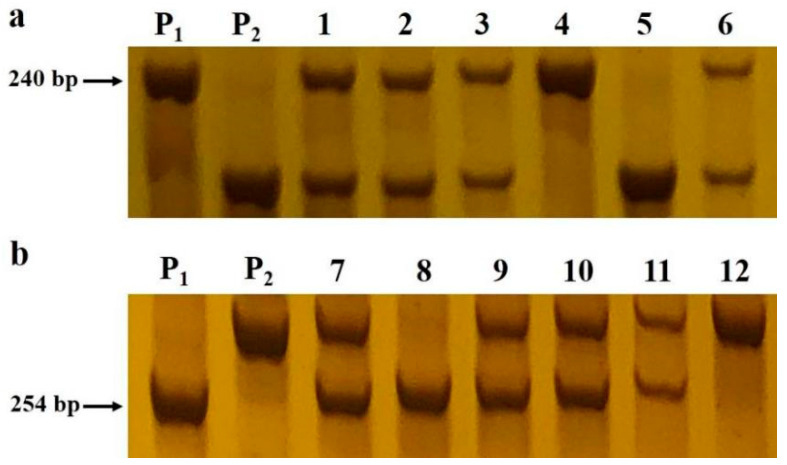
Experimental results of polymorphic markers Indel-78 and Indel-15. Marker scan with progeny BC_5_F_3_ and BC_6_F_2_ populations derived from the parents ZS11 and NJAU5773 was conducted. P_1_ and P_2_ indicates PCR products from the parents ZS11 and NJAU5773 plants, respectively. (**a**) Shows partial results for InDel marker Indel-78 in which 1–3 and 6 denote the PCR products from the heterozygous plants in the progeny populations, and 4 and 5 denotes products from the homozygous plants with flatten leaves and upright siliques and plants with up-curling leaves and downward-pointing siliques, respectively. (**b**) Shows partial results for InDel marker Indel-15 in which 7 and 9–11 denote the PCR products from the heterozygous plants in the progeny populations, and 8 and 12 denotes products from the homozygous plants with flatten leaves and upright siliques and plants with up-curling leaves and downward-pointing siliques, respectively.

To more precisely map the *BnUD1* locus, 49 primer pairs for InDel markers were designed to uniformly cover the preliminary mapping interval on the basis of the BSA sequencing results. Fifteen markers targeting the smaller candidate region on the A05 chromosome (37,261,266–37,670,607 bp) were polymorphic (i.e., ID-62, ID-63, ID-64, ID-65, ID-66, ID-67, ID-68, ID-70, ID-73, ID-74, ID-77, ID-78, ID-79, ID-81, and ID82) (Table S3), whereas 12 polymorphic InDel markers were obtained for the larger candidate region on the A05 chromosome (7,094,487–11,086,188 bp; i.e., InDel-2, InDel-3, InDel-5, InDel-6, InDel-7, InDel-8, InDel-9, InDel-11, InDel-12, InDel-13, InDel-15, and InDel-16) (Figure 2a; Table S3). These polymorphic markers were used to genotype 1042 individuals in the BC_5_F_3_ and BC_6_F_2_ populations. The mapping using the JoinMap 4.1 software indicated that the *BnUD1* locus was located in a mapping interval between InDel-13 and InDel-15 (1.63 Mb) on the A05 chromosome (Figure 2a), which eliminated one of the candidate regions (37,261,266–37,670,607 bp). To fine map the *BnUD1* locus, 29 InDel marker primers were designed to target the narrowed mapping interval. Of these markers, 10 were polymorphic (InDel-47, InDel-50, InDel-55, InDel-56, InDel-57, InDel-65, InDel-67, InDel-70, InDel-72, and InDel-73). The analysis of the segregating population using these polymorphic markers generated a 466.58 kb mapping interval (Figure 2b), which was used to develop four InDel markers (InDel-75, InDel-78, InDel-80, and InDel-81). With these four markers, the *BnUD1* locus was mapped to a 54.84 kb interval between InDel-78 and InDel-15 (Figure 2c and Figure 3). The other markers developed according to the BSA sequencing results were unable to further narrow the mapping interval.

### 2.4. Gene Cloning

**Figure 4:**
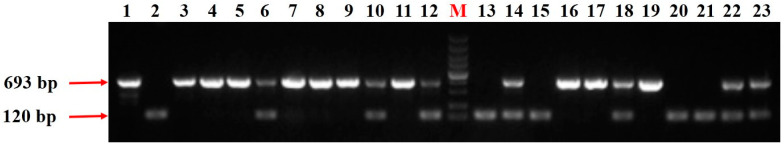
Partial molecular marker experimental results of the InDel marker BnA05ID1. ‘M’ denotes 5000 marker. The number 1, 3–5, 7–9, 11, 16–17, and 19 denote the PCR products from homozygous plants with up-curling leaf and downward-pointing silique. The number 6, 10, 12, 14, 18, 22, and 23 denote the PCR products from heterozygous plants with up-curling leaf and downward-pointing silique. The number 2, 13, 15, 20, and 21 denote the PCR products from homozygous plants with flatten leaf and upright silique.

The homologous segment sequences in the fine mapping interval were downloaded from the *B. napus* cv. ZS11 genome database. The genes in the interval were annotated on the basis of the *A. thaliana* genome to identify the candidate genes associated with the up-curling leaf and downward-pointing silique traits. The interval harbored 11 annotated genes (Table 2). To analyze the candidate genes in the *BnUD1* locus, we cloned the 11 genes from the two parents of the mapping populations (NJAU5773 and ZS11). The subsequent alignment of these sequences indicated that *BnaA05G0157900ZS*, *BnaA05G0158100ZS*, and *BnaA05G0158300ZS* differed between the two parents (Figure S2), whereas the other examined genes within the mapping interval were identical in the two parents. The comparison of the *BnaA05G0157900ZS* sequences revealed that the encoded protein in NJAU5773 has three amino acid (AA) substitutions, of which the Gly-to-Ala mutation (G45A) occurred in the trans-membrane region, the Gly-to-Ser mutation (G122S) was detected in the PMEI domain, and the Gly-to-Asp mutation (G394D) was present in the pectinesterase domain (Figure S2a). In addition, a 573 bp segment was inserted into the pectinesterase domain of *BnaA05G0157900ZS* gene in the *Bnud1* mutant. Next, we developed an InDel marker (BnA05ID1) specific for this insertion (Table S3). The marker co-segregated with the up-curling leaf and downward-pointing silique phenotypes in the segregating populations (Figure 4). These sequence alterations may affect leaf and silique trait formation in the *Bnud1* mutant.

The protein encoded by *BnaA05G0158100ZS* in the *Bnud1* mutant included one substitution (Val-to-Asp) at AA position 146, which is in the RING domain of a RING/U-box superfamily protein (Figure S2b). This substitution may lead to a change in function. The examination of *BnaA05G0158300ZS* in the *Bnud1* mutant detected three AA substitutions, but they were not located in the IENR2 domain encoded specifically by this gene (Figure S2c). The results of the sequence analyses suggested that *BnaA05G0157900ZS* and *BnaA05G0158100ZS* are the most likely candidate genes in the *BnUD1* locus.

### 2.5. Candidate Gene Analysis

To determine whether the gene mutations are associated with the mutant traits, we further examined the gene functions by conducting a bioinformatic analysis as well as gene expression experiments using a pair of isogenic lines with the ZS11 genetic background. The *BnaA05G0157900ZS* gene is homologous to *AT1G53840*, which encodes PECTIN METHYLESTERASE 1 (PME1). The PMEs can catalyze the specific demethylesterification of HG to form load-bearing Ca^2+^ crosslinks that affect the texture and rigidity of the cell wall [29,30,31]. Thus, they are reportedly involved in various biological processes, including cell wall expansion, seed germination, and hypocotyl elongation [45,46,47]. The overexpression of PMEI-encoding genes in wild-type *A. thaliana* plants leads to the production of curled leaves, a convoluted shoot, and downward-pointing siliques [34,35]. In the present study, the four sequence changes in the *Bnud1* mutant *BnaA05G0157900ZS* gene were located in the gene-coding region or intron region. On the basis of the previously reported potential function of the protein encoded by BnaA05G0157900ZS, these sequence mutations are probably responsible for the observed mutated traits.

The mutation in the *Bnud1* mutant *BnaA05G0158100ZS* gene was located in the sequence encoding the RING/U-box domain. This gene is homologous to *AT1G53820*, which encodes the biological stress-responsive RING/U-box superfamily member ARABIDOPSIS TOXICOS EN LEVADURA 60 (AL60). This gene has not been linked to substantial alterations to leaf and silique morphological characteristics [48,49,50,51,52]. The *BnaA05G0158300ZS* gene in *Bnud1* has three mutations that are not within the sequence encoding the conserved IENR2 domains. Moreover, *BnaA05G0158300ZS* is homologous to *AT1G53800*, which was annotated as a gene involved in sarcomeric titin assembly during cardiac myofibrillogenesis in animals [53,54]. However, *AT1G53800* homologs in plants have not been investigated.

**Figure 5:**
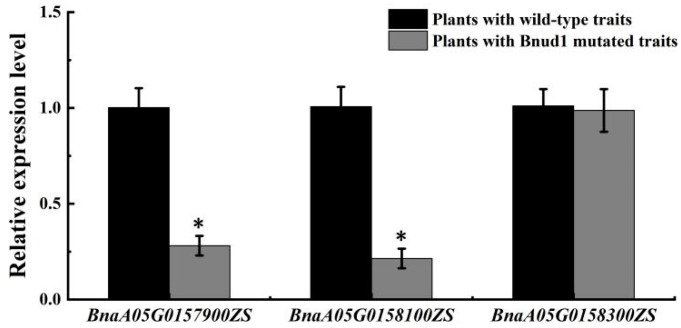
Differential expression of 3 mutated genes in the mapping interval in leaves of the advanced backcross population. The Actin of *B. napus* was used as the reference gene for normalization in qRT-PCR experiments. The relative mRNA level of genes in plants with *Bnud1* mutated traits (grey) was calculated in reference to gene expression level of plants with wild-type traits (black). Values shown are means ± SD (*n* = 4). * Denotes significant at probability level of 0.05.

The expression levels of the three mutated genes in the mapping interval were determined by performing a quantitative real-time polymerase chain reaction (qRT-PCR) analysis of leaf samples from the four plants with the *Bnud1* mutated traits and the four plants with the wild-type traits in the BC_6_F_2_ population. Both *BnaA05G0157900ZS* and *BnaA05G0158100ZS* were expressed at significantly lower levels in the plants with the *Bnud1* mutated traits than in the plants with the wild-type traits (Figure 5). In contrast, the *BnaA05G0158300ZS* expression level did not differ between the two plant types. This implied that *BnaA05G0158300ZS* is not associated with the formation of mutated traits (Figure 5). These results suggested that *BnaA05G0157900ZS* and *BnaA05G0158100ZS* may be responsible for the up-curling leaf and downward-pointing silique traits of the *Bnud1* mutant.

### 2.6. Agronomic Traits

To evaluate the effects of the *BnUD1* locus on plant agronomic traits, 30 plants with the *Bnud1* mutated traits and 30 plants with the wild-type traits were randomly sampled from the BC_6_F_2_ population derived from the cross between NJAU5773 and the recurrent parent ZS11. The values for some of the agronomic traits, including plant height, branch height, main inflorescence length, number of first effective branches, and 1000-seed weight, were significantly lower for the plants with up-curling leaves and downward-pointing siliques than for the plants with flat leaves and upright siliques (Table 3). The other agronomic traits, including stem diameter, siliques of the main inflorescence, total siliques per plant, and silique length, did not differ between the plants with and without the *BnUD1* locus (Table 3). Thus, the *BnUD1* locus appeared to negatively affect the plant stature, resulting in a compact architecture. Additionally, the number of seeds per silique was significantly higher for the plants with the *BnUD1* locus than for the plants without the *BnUD1* locus, reflecting the positive effects of the *BnUD1* locus on the seed yield.

### 2.7. Determination of the Chlorophyll Content and Photosynthetic Efficiency

Compared with the plants with the wild-type traits at the seedling stage, the leaf chlorophyll (Chl) a, Chl b, and total Chl contents as well as the Chl a/b ratio were significantly higher for the plants with the *Bnud1* mutated traits selected from the BC_6_F_2_ population derived from the cross between the NJAU5773 and the recurrent parent ZS11 (Table 4). This result indicated that the *Bnud1* mutated traits were associated with increases in the leaf Chl content. The leaf net photosynthetic rate, stomatal conductance, and concentration of intercellular CO_2_ were significantly higher in the plants with the *BnUD1* locus than in the plants without the *BnUD1* locus. However, the leaf transpiration rate did not differ between the plants with the *BnUD1* locus and the plants without the *BnUD1* locus (Table 5). Hence, the *BnUD1* locus may lead to increased photosynthetic efficiency.

## 3. Discussion

Leaves and siliques are important photosynthesis-related organs that influence the agronomic value of crops. Up-curling leaves and downward-pointing siliques are typically the result of the heteromorphic development of plant organs. However, the genetic mechanism underlying the formation of up-curling leaves is generally unrelated to the genetic basis of silique formation.

There has recently been some progress in the research on the genes related to leaf curling in *A. thaliana* and rice [1,6,7,9,55]. For example, the MYB transcription factor-encoding *PHANTASTICA* (*PHAN*) gene was first reported to be associated with leaf adaxial-abaxial polarity in *Antirrhinum majus* [56,57]. In *A. thaliana*, AS1 is homologous to PHAN and regulates leaf polarity by forming a protein complex with the plant-specific lateral organ boundaries (LOB) family protein AS2 [11,55]. Several transcription factors (e.g., HD-ZIP III, KAN, and YABBY) can bind to the *AS1* gene promotor to regulate the establishment of leaf abaxial polarity in *A. thaliana*, resulting in leaf-rolling behavior [4,58,59,60,61,62]. Auxin response factors, such as ARF3, ARF4, and ARF2, can form a complex with KAN to modulate leaf abaxial polarity [12,63,64]. MicroRNAs, including miR165 and miR166 targeting HD-ZIP III [62,65] and miR390 triggering the production of phasi-RNAs from TAS3 trans-acting short interfering RNA transcripts, also influence leaf-rolling behaviors by repressing ARF activities [66,67].

Silique morphological development does not generally depend on leaf developmental genes. Previous research on silique formation focused on silique yield-associated traits, such as seed number and weight within siliques and silique length. Some genes regulating silique development have been identified, including the silique length-related gene *REPLUMLESS* (*RPL*) [68], silique shattering-related genes *SHATTERPROOF1* (*SHP1*) and *SHP2* [69], *INDEHISCENT* (*IND*) [70], *ALCATRAZ* (*ALC*) [71], and silique polarity-associated *KNOX* genes [27]. To date, how silique angles form remains unknown. In *A. thaliana*, the *KNOX* gene *BREVIPEDICELLUS* (*BP*), which is expressed downstream of the auxin and AS1 pathways, may encode a protein that regulates silique polarity signals by inhibiting auxin transport, thereby affecting silique and pedicel morphological development [27,72].

Mutations that alter the ability of PMEs to catalyze the demethylesterification of the cell wall HG may modify cell wall components, resulting in up-curling leaves and silique and stem morphological abnormalities [29,34,35]. In this study, a *PME* gene contributing to the up-curling leaf and downward-pointing silique traits was identified (Figure 2 and Figure S2a). More specifically, the marker analysis revealed that mutations in the *PME* gene *BnaA05G0157900ZS* may help to explain the formation of downward-pointing siliques and up-curling leaves (Figure 3 and Figure 4). Another candidate gene (*BnaA05G0158100ZS*) for the mutated traits encodes a RING/U-box-containing protein associated with biological stress responses (Table 2). Although this gene may be part of a complex genetic regulatory system, whether it is directly associated with the mutated traits is unclear.

The mutant NJAU5773, which was originally identified in a breeding population and obtained via consecutive generations of selfing, has three mutated genes in the mapping interval (Figure S2). When the two candidate genes for the *BnUD1* locus-associated traits were used as queries to screen for the corresponding gene sequences in the *B. napus* pan-genome database (http://cbi.hzau.edu.cn/bnapus/), the mutated candidate genes were undetectable in other genomes. The segment harboring the *BnUD1* locus is located in the A sub-genome of *B. napus*, similar to the homologous segment in the A genome of *B. rapa*. However, the candidate genes differed from the corresponding homologous genes. Therefore, the candidate gene mutations are unique to NJAU5773 and may be the product of natural evolutionary events.

Moderately up-curled leaves and downward-pointing siliques can theoretically increase light transmittance and the light saturation point, thereby increasing the overall photosynthetic efficiency. The increased planting density associated with appropriately up-curled leaves and downward-pointing siliques may also positively affect the harvest index [73,74]. Thus, the up-curling leaf trait should be explored in more detail. To date, three loci related to the up-curling leaf trait have been identified in *B. napus* (i.e., *BnUC1*, *BnUC2*, and *BnUC3*) [39,40,41]. In present work, we found that the *Bnud1* mutant had an increased photosynthetic efficiency at seedling stage, and relatively small plant architecture (Figure 1; Table 5). The *BnUD1* locus is expected to be applied to increase the efficiency of leaf photosynthetic activities and decrease plant height (Table 3 and Table 5), but the underlying mechanisms will need to be characterized in future investigations. Unlike the other loci mediating the up-curling of leaves, the *BnUD1* locus also controls the formation of downward-pointing siliques and increases in the number of seeds per silique (Table 3). However, the reason for the increase in the number of seeds per silique is unclear. We speculate that the influx of carbon is enhanced in the downward-pointing siliques. Alternatively, the increase in seed production may be the result of increased pollination efficiency.

## 4. Materials and Methods

### 4.1. Plant Materials

The double-low *B. napus* (oilseed rape) line NJAU5773 with up-curling leaves (before the budding stage) and downward-pointing siliques obtained from our germplasm and canola variety ZS11 provided by Nanjing Agricultural University were used as the parents to produce the F_1_ population. The F_1_ individuals were selfed to generate F_2_ mapping populations and backcrossed with the recurrent parent ZS11 (female) to construct the mapping populations. The selfed and backcrossed populations were examined to calculate the segregation ratio of plants with up-curling leaves and downward-pointing siliques to plants with flat leaves and upright siliques. The BC_5_F_3_ and BC_6_F_2_ populations were used for the preliminary and fine mapping of the *BnUD1* locus. The plants with up-curling leaves and downward-pointing siliques and the plants with flat leaves and upright siliques in the BC_6_F_2_ population were used for the BSA, qRT-PCR, and analyses of the Chl content, photosynthetic efficiency, and agronomic traits.

All materials were grown on the research farm of Nanjing Agricultural University (Nanjing, China). Plants were cultivated in 2.5 m rows, with 15 plants per row and 0.4 m between rows.

### 4.2. Genetic Analysis

To determine the number of genes controlling the up-curling leaf and downward-pointing silique phenotype of the *Bnud1* mutant, all generations, including the F_1_, F_2_, and BC_1_ populations and the derived lines (BC_1_-BC_6_, BC_5_F_3_, and BC_6_F_2_), were grown in the field. Leaf and silique morphology were assessed at the seedling and maturity stages, respectively. Chi-square tests were performed using the segregation data in each population to analyze the genetic regulation of the up-curling leaf and downward-pointing silique traits.

### 4.3. Bulked Segregant Analysis

To map the *BnUD1* locus, a BC_6_F_2_ population was developed from the cross between the *Bnud1* mutant with up-curling leaves and downward-pointing siliques and wild-type plants with flat leaves and upright siliques. Genomic DNA was extracted from young leaves using cetyl-trimethylammonium bromide (CTAB). The BC_6_F_2_ family population was identified according to a BSA. Equal amounts of DNA from 30 plants with up-curling leaves and downward-pointing siliques and 30 wild-type plants from the BC_6_F_2_ population were pooled to form the *Bnud1* mutant trait bulk (UDB) and the wild-type trait bulk (WTB), respectively. The polymorphisms between the bulks (UDB and WTB) were screened using InDel markers from a previous study [75].

To efficiently develop linked markers in the target region in *B. napus*, a BSA sequencing (BSA-seq) experiment was performed. Genomic DNA from the different bulks was subjected to a whole-genome sequencing analysis. Short-insert (350–450 bp) sequencing libraries were constructed from approximately 2 μg parental genomic DNA using the TruSeq^®^ DNA Sample Preparation Kit (Illumina, San Diego, CA, USA). The quantified libraries were sequenced on the HiSeq 3000 platform (Illumina) to produce 150 bp paired-end reads. The InDels and SNPs were called as previously described [76,77]. The de novo assembled ZS11 genome was used as the reference for calculating the SNP index of UDB and WTB. The ΔSNP index was calculated by subtracting the SNP index for UDB from the SNP index for WTB.

### 4.4. Mapping of the BnUD1 Locus

The BSA-seq results for the mapping interval were used to identify 5345 SNPs/1275 InDels and 721 SNPs/199 InDels covering 3.99 Mb and 0.41 Mb intervals between UDB and WTB (Figure S1; Table S2). Next, 103 differential InDel sites (at positions 37,261,266–37,670,607 bp and 7,094,487–11,086,188 bp of A05) were used to develop molecular markers that could narrow the mapping interval. The InDel markers were used to analyze the BC_5_F_3_ and BC_6_F_2_ populations to fine map the *BnUD1* locus (Table S3). Finally, a fine linkage map for the locus associated with the up-curling leaf and downward-pointing silique traits was constructed using the JoinMap 4.1 software and the polymorphic InDel markers [78]. The primers used are listed in Table S3.

The PCR conditions for the molecular marker experiments were as follows: 94 °C for 5 min; 35 cycles of 94 °C for 30 s, annealing temperature of each InDel marker for 30 s, and 72 °C for 30 s; 72 °C for 10 min.

### 4.5. Identification of Genes in the Mapping Interval and Comparative Sequencing

The sequences of the fine mapping interval on the *B. napus* cv. ZS11 A05 chromosome were downloaded from the Brassicaceae database (http://brassicadb.org/brad/, accessed on 15 January 2023) and the *B. napus* pan-genome information online resource (http://cbi.hzau.edu.cn/bnapus/, accessed on 15 January 2023) to identify the genes in the mapping interval. The genes detected in the mapping interval were annotated on the basis of *B. napus* cv. ZS11 annotated genes.

Extracted DNA was digested using RNase I (Takara, Dalian, China) to remove RNA. The RNA-free DNA samples were genotyped by genome sequencing. Total RNA was extracted from the leaves at the seedling stage using the RNAprep Pure Plant Kit (BioTeke, Beijing, China). First-strand cDNA was synthesized from the RNA using a reverse transcription kit (Takara, Tokyo, Japan). All 11 genes identified in the mapped interval were cloned from the two parents with gene-specific primers designed using the Primer Premier 5.0 software [79] (Tables S4 and S5). The PCR amplifications were performed as previously described [80]. The amplified fragments were inserted into the pEASY-Blunt Cloning Kit vector (TransGen, Beijing, China) and sequenced. The resulting sequences were aligned using Clustal X1.83 software [81].

### 4.6. Quantitative Real-Time PCR Analysis

A qRT-PCR analysis was conducted to compare the expression levels of the three mutated genes in the mapping interval between the plants with the *Bnud1* mutated traits and the plants with the wild-type traits in the BC_6_F_2_ population. Leaves were collected from four plants at the five-leaf stage and then used to prepare the cDNA template for the qRT-PCR analysis, which was completed with gene-specific primers (Table S6). The gene expression levels were normalized against the expression of an BnActin gene (i.e., housekeeping gene) (Table S6). The qRT-PCR was performed using the SYBR Green Real-time PCR Master mix and the CFX96-2 PCR system (Bio-Rad, Hercules, CA, USA) and the relative expression levels were analyzed as described previously [82]. Relative expression levels were calculated according to the 2^−ΔΔCt^ method, with the actin gene serving as the internal control. Four biological replicates were used.

### 4.7. Agronomic Trait Analysis

To investigate the effects of the *BnUD1* locus on plant agronomic traits, 30 plants with the *Bnud1* mutated traits and 30 plants with the wild-type traits were randomly selected from the BC_6_F_2_ population. The examined agronomic traits included plant height, branch height, main inflorescence length, stem diameter, number of first effective branches, number of siliques on the main inflorescence, total number of siliques per plant, silique length, seeds per silique, and 1000-seed weight. The mean values for all agronomic traits were compared between the plants with *Bnud1* mutated traits and the plants with wild-type traits by *t*-tests.

### 4.8. Determination of the Chlorophyll Content and Photosynthetic Efficiency

Fifteen homozygous plants with the *Bnud1* mutated traits and 15 plants with the wild-type traits were randomly selected from the BC_6_F_2_ population at the seedling stage to measure the Chl contents. Specifically, Chl was extracted from 0.2 g fresh leaves using 50 mL 80% acetone, after which the Chl content was determined using the Alpha-1500 spectrophotometer (LASPEC, Shanghai, China). The leaf Chl a, Chl b, and total Chl contents were measured as previously described [83,84].

Six plants with the *BnUD1* locus and six plants without the *BnUD1* locus were randomly selected from the BC_6_F_2_ population at the seedling stage for an analysis of photosynthetic efficiency. The photosynthetic characteristics of the plants were determined using the Li-Cor 6400 portable photosynthesis system (Li-Cor Inc., Lincoln, NE, USA), with the built-in light source set at 1000 μmol photons m^−2^ s^−1^ at 23 °C as previously described. All measurements were completed between 9:00 a.m. and 11:00 a.m. [84].

## 5. Conclusions

A new mutant *B. napus* plant (NJAU5773) with up-curling leaves and downward-pointing siliques was identified in our *B. napus* germplasm. Inheritance studies showed that the up-curling leaf and downward-pointing silique traits were controlled by one dominant locus, which was mapped to a 54.84 kb interval on the BnA05 chromosome using BSA-seq and InDel markers. Both *BnaA05G0157900ZS* and *BnaA05G0158100ZS* were identified as candidate genes on the basis of sequencing and gene expression analyses. The examination of individual plants revealed the *BnUD1* locus had positive effects on photosynthetic efficiency. In conclusion, the findings of this study provide a theoretical foundation for elucidating the mechanism mediating the up-curling leaf and downward-pointing silique traits, which may be relevant for *B. napus* breeding programs.

## Figures and Tables

**Table 1:** Inheritance of the leaf up-curling and silique downward-pointing traits in populations derived from the two parents in *B. napus*.

Population	UD	WT	Total	Expectation	$\chi^{2}$
F_1_	30	0	30		
RF_1_	30	0	30		
F_2_	100	42	142	3:1	1.43
BC_1_	58	70	128	1:1	1.13
BC_2_	59	73	132	1:1	1.48
BC_3_	55	65	120	1:1	0.83
BC_4_	63	53	116	1:1	0.86
BC_5_	74	60	134	1:1	1.46
BC_6_	53	65	118	1:1	1.22
BC_5_F_3_	527	207	734	3:1	3.56
BC_6_F_2_	220	88	308	3:1	1.91

UD denotes plants with up-curling leaf and downward-pointing silique plants; WT denotes that with flatten leaves and upright siliques.

**Table 2:** Genes in the mapped interval and the *Brassica* within the interval on the A05 chromosome.

Gene in *B. napus.* cv ZS11	Gene in *B. napus.* cv Darmor	Homologue in *A. thaliana*	Gene Function
*BnaA05G0157300ZS*	*BnaA05g14090D*	*AT2G20619*	Plant thionin family protein
*BnaA05G0157400ZS*			Unknown
*BnaA05G0157500ZS*		*AT4G18570*	Tetratricopeptide repeat (TPR)-like superfamily protein
*BnaA05G0157600ZS*		*AT4G18570*	Tetratricopeptide repeat (TPR)-like superfamily protein
*BnaA05G0157700ZS*	*BnaA05g14100D*	*AT1G53860*	Remorin family protein
*BnaA05G0157800ZS*	*BnaA05g14110D*	*AT1G53850*	20S proteasome alpha subunit E1
*BnaA05G0157900ZS*	*BnaA05g14120D*	*AT1G53840*	Pectin methylesterase 1
*BnaA05G0158000ZS*	*BnaA05g14130D*	*AT1G53830*	Pectin methylesterase 2
*BnaA05G0158100ZS*	*BnaA05g14140D*	*AT1G53820*	RING/U-box superfamily protein
*BnaA05G0158200ZS*	*BnaA05g14150D*	*AT5G38830*	Cysteinyl-tRNA synthetase, class Ia family protein
*BnaA05G0158300ZS*	*BnaA05g14160D*	*AT1G53800*	Muscle M-line assembly protein

**Table 3:** Agronomic traits comparison between plants with *BnUD1* locus and that without *BnUD1* locus in the BC_6_F_2_ population.

Trait	Plants without *BnUD1* Locus	Plants with *BnUD1* Locus
Plant height (cm)	180.76 ± 1.95	144.61 ± 6.49 *
Branch height (cm)	56.86 ± 5.03	33.70 ± 4.55 *
Main inflorescence length (cm)	73.94 ± 2.44	66.50 ± 8.41 *
Stem diameter (mm)	24.37 ± 1.85	23.10 ± 0.66
Number of first effective branch	8.80 ± 0.84	6.88 ± 0.99 *
Siliques of main inflorescence	72.63 ± 6.78	71.60 ± 4.45
Total siliques per plant	346.20 ± 18.83	363.13 ± 29.86
Silique length	9.23 ± 0.63	9.37 ± 0.78
Seeds per siliques	26.94 ± 1.94	30.67 ± 2.54 *
1000-seed weight (g)	5.42 ± 0.18	4.13 ± 0.15 *

* Indicates significant at the 0.05 probability level by *t*-test. Data are shown as mean ± SD (*n* = 30 for each sample).

**Table 4:** Leaf chlorophyll contents in leaves of plants with *BnUD1* locus and that without *BnUD1* locus.

Genotype	Chl a (mg/g)	Chl b (mg/g)	Total	Chl a/b Ratio
Plants without *BnUD1* locus	1.59 ± 0.33	0.96 ± 0.32	2.55 ± 0.62	1.75 ± 0.37
Plants with *BnUD1* locus	3.39 ± 0.36 *	1.80 ± 0.23 *	5.19 ± 0.57 *	1.89 ± 0.27 *

* Indicates significant at the 0.05 probability level. Mean ± standard deviation (SD) under sample size. (*n* = 15 for each sample).

**Table 5:** Photosynthetic indicators in leaves of plants with *BnUD1* locus and that without *BnUD1* locus.

Genotype	NPR µmol CO_2_ m^−2^ s^−1^	SC mol H_2_O m^−2^ s^−1^	ICC µmol CO_2_ mol^−1^	TR mmol H_2_O m^−2^ s^−1^
Plants without *BnUD1* locus	8.96 ± 0.48	0.24 ± 0.03	368.86 ± 4.82	2.46 ± 0.31
Plants with *BnUD1* locus	11.89 ± 0.76 *	0.42 ± 0.02 *	425.50 ± 6.37 *	2.89 ± 0.37

Data are presented as means ± SD. * Indicates significant at 0.05 probability level. NPR, SC, ICC, and TR denotes net photosynthetic rate, stomatal conductance, intercellular CO_2_ concentration and transpiration rate, respectively. (*n* = 6 for each sample).

## Data Availability

The datasets generated during and/or analyzed during the current study are available from the corresponding author upon reasonable request.

## Supplementary Materials

The following supporting information can be downloaded at: https://www.mdpi.com/article/10.3390/ijms24043069/s1.
