# Supplementary material for: Fine Mapping of a Pleiotropic Locus (BnUD1) Responsible for the Up-Curling Leaves and Downward-Pointing Siliques in Brassica napus
Source: Int J Mol Sci. 2023 Feb 4;24(4):3069. doi: 10.3390/ijms24043069 (PMC9965582; doi:10.3390/ijms24043069)
Supplement: Supplementary file 1 [file ijms-24-03069-s001.zip › ijms-2146867-supplementary.pdf]

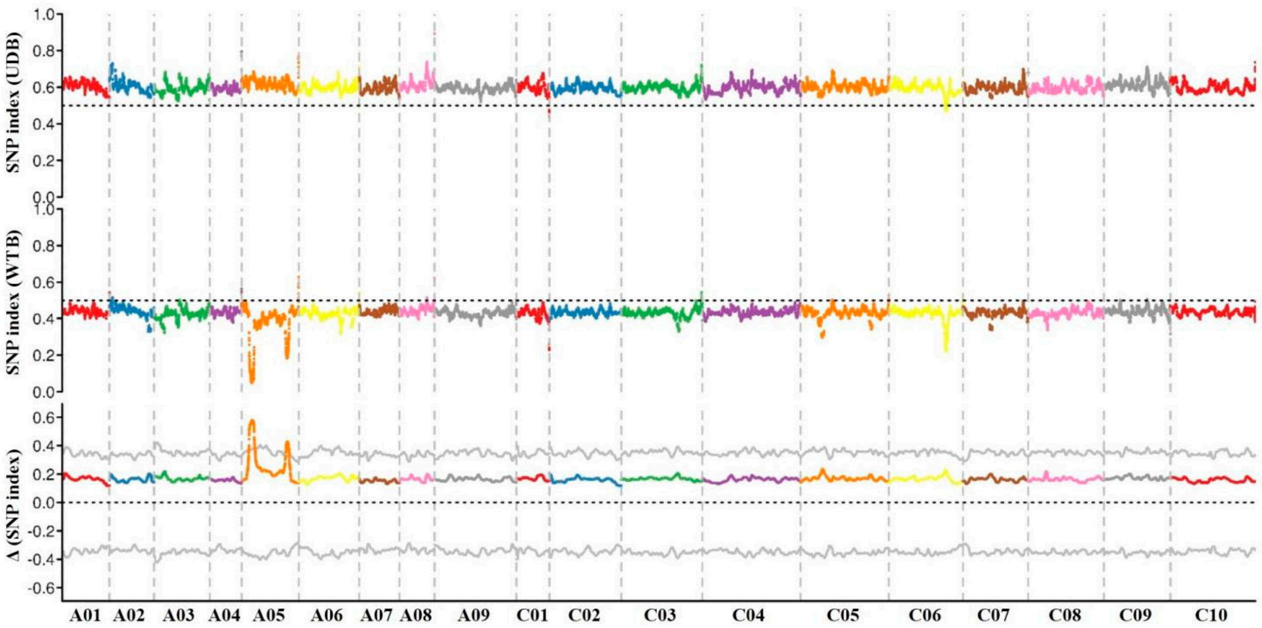


**Figure S1.** The distribution of the Δ (SNP index) values on 19 chromosomes in *Brassica napus*. The horizontal axis represents the physical position (Mb) of the 19 chromosomes in *Brassica napus*. The vertical axis represents the SNP index, which was estimated according to a 2 Mb sliding window. The Δ (SNP index) was plotted by subtracting the UDB from the WTB. The horizontal gray line is the threshold dotted line for Δ (SNP index) ($P$=0.009).

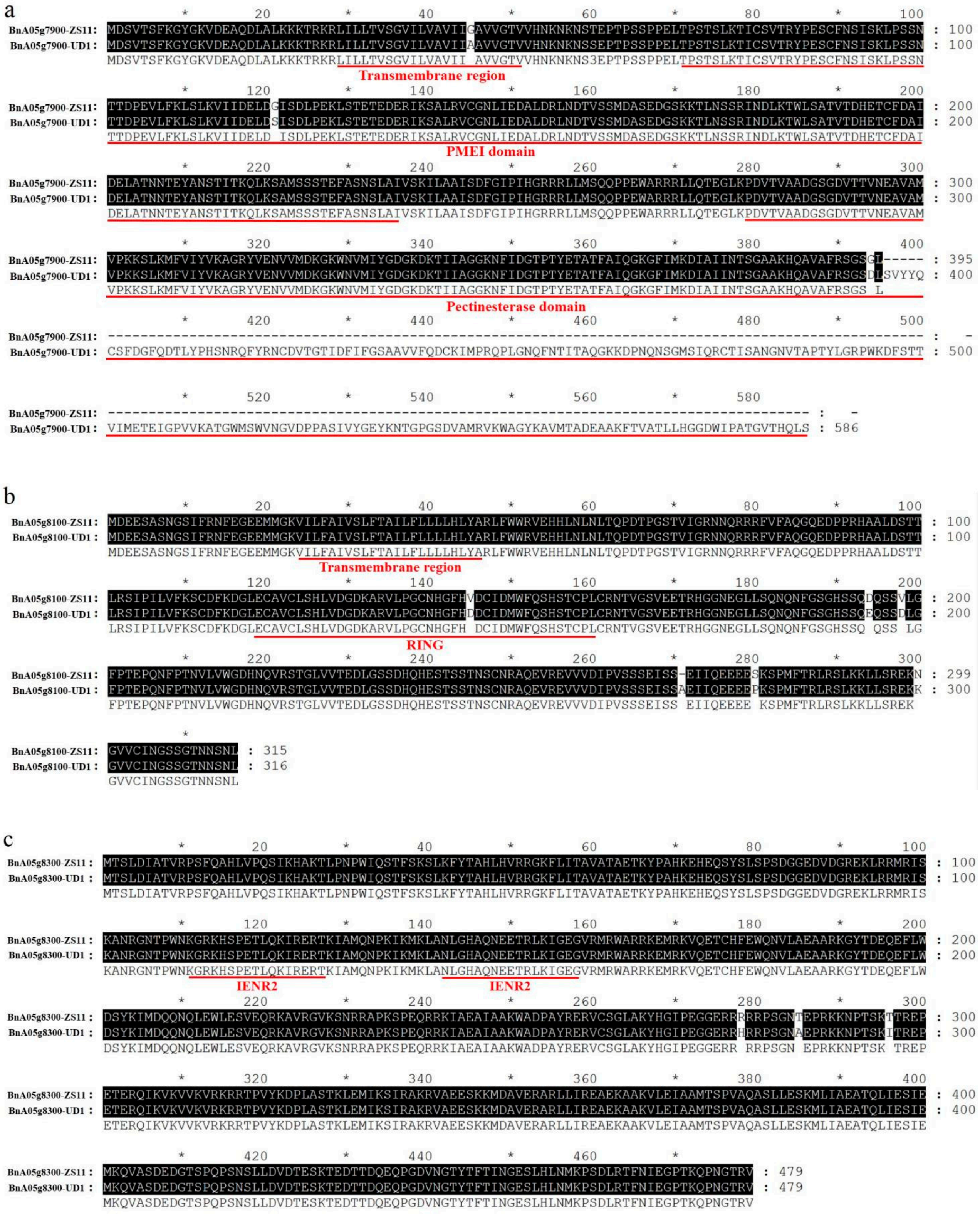


**Figure S2.** Amino acid sequence alignment of BnaA05G0157900ZS, BnaA05G0158100ZS, and BnaA05G0158300ZS in ZS11 and *Bnud1* plants. **a** The amino acid sequence alignment of BnaA05G0157900ZS in ZS11 and *Bnud1* plants. **b** The amino acid sequence alignment of BnaA05G0158100ZS in ZS11 and *Bnud1* plants. **c** The amino acid sequence alignment of BnaA05G0158300ZS in ZS11 and *Bnud1* plants. * denotes the advance of 10.

**Table S1.** Distribution of SNP loci and InDel sites between UDB and WTB on 19 chromosomes.

| Chr | Length | No. SNPs | SNP Density | No. InDels | InDel Density |
|---|---|---|---|---|---|
| A01 | 38,004,428 | 20,426 | 537.46 | 4,997 | 131.48 |
| A02 | 35,943,954 | 12,213 | 339.78 | 2,534 | 70.50 |
| A03 | 44,868,710 | 23,947 | 533.71 | 5,895 | 131.38 |
| A04 | 25,679,024 | 9,065 | 353.01 | 1,631 | 63.51 |
| A05 | 45,991,561 | 21,126 | 459.35 | 3,981 | 86.56 |
| A06 | 48,704,706 | 16,278 | 334.22 | 2,504 | 51.41 |
| A07 | 32,302,721 | 14,637 | 453.12 | 2,822 | 87.36 |
| A08 | 28,329,074 | 7,489 | 264.36 | 1,394 | 49.21 |
| A09 | 65,862,748 | 20,277 | 307.87 | 3,193 | 48.48 |
| A10 | 26,592,803 | 10,020 | 376.79 | 1,922 | 72.28 |
| C01 | 57,880,920 | 39,774 | 687.17 | 7,943 | 137.23 |
| C02 | 65,293,782 | 25,284 | 387.23 | 3,472 | 53.18 |
| C03 | 79,061,710 | 43,464 | 549.75 | 8,758 | 110.77 |
| C04 | 71,179,181 | 27,009 | 379.45 | 3,886 | 54.59 |
| C05 | 59,550,008 | 31,883 | 535.40 | 5,171 | 86.83 |
| C06 | 52,512,057 | 24,737 | 471.07 | 3,859 | 73.49 |
| C07 | 60,986,212 | 29,408 | 482.21 | 4,029 | 66.06 |
| C08 | 53,660,391 | 20,271 | 377.76 | 2,875 | 53.58 |
| C09 | 68,416,614 | 47,211 | 690.05 | 9,026 | 131.93 |
| Total | 960,820,604 | 444,519 | 462.65 | 79,892 | 83.15 |

**Table S2.** The candidate region information on A05 chromosome.

| Chromosome | Start (bp) | End (bp) | Length (Mb) | No. Genes |
|---|---|---|---|---|
| A05 | 7,094,487 | 11,086,188 | 3.99 | 512 |
| A05 | 37,261,266 | 37,670,607 | 0.41 | 64 |

**Table S3.** The designed primers of polymorphic InDel markers used in this study.

| Name of Primers | Sequence of Primers | Product Length | Chromosome Location (bp) |
|---|---|---|---|
| InDel-2-F | TTAGAAATGACTTGTGGCT | 220 bp | 8042178 |
| InDel-2-R | GATAAGACAGGTCTGAGGC | | |
| InDel-3-F | TATCCCAAATAACCCAATC | 251 bp | 8174210 |
| InDel-3-R | CTTCTCAAATCAAGAGCAAT | | |
| InDel-5-F | AAAGAAGTCGAGGAAGGTG | 235 bp | 8272771 |
| InDel-5-R | AGTATTTGTTTCGTTTCAGATC | | |
| InDel-6-F | ATTTAGCATCGTCCTCTTCGC | 218 bp | 8298485 |
| InDel-6-R | CATCGTCTCACGCCCGTA | | |
| InDel-7-F | TGCTTGGAGGAGTTTTACA | 211 bp | 8300248 |
| InDel-7-R | CAACATCAGGTGCCATTAG | | |
| InDel-8-F | GAACACTCCTCGCTTCATT | 286 bp | 8317457 |
| InDel-8-R | TTCCTAACTTCTGCCACCT | | |
| InDel-9-F | ATCAGGCAAAGGAAGATTT | 250 bp | 8449755 |
| InDel-9-R | AAAAAGGGTCTGGAGTCATA | | |
| InDel-11-F | ATCTTAGACGGGAGCGAGTT | 214 bp | 8455341 |
| InDel-11-R | TTCGCAACATCAGGCAAAC | | |
| InDel-12-F | TGCCATAGTTCCGCATTCC | 234 bp | 8458842 |
| InDel-12-R | CAGCCCTAACTCAGACTTTACCC | | |
| InDel-13-F | AATACAATTAGTTATACGACGAC | 247 bp | 8482656 |
| InDel-13-R | AAATGAATCAACAACCAAAC | | |
| InDel-15-F | TACTAGCCTTCCACGAACC | 254 bp | 10110233 |
| InDel-15-R | TCAAGAACAACCAGGAGATG | | |
| InDel-16-F | CAAAAGGAAGAAGGCACA | 200 bp | 10111608 |
| InDel-16-R | CAAAACCCAAAGGTGATG | | |
| InDel-47-F | TAGTTCCCTTCATATTCCA | 234 bp | 8535970 |
| InDel-47-R | CTTCATCACCATCTTCATC | | |
| InDel-50-F | GTAACCCTTGACTCTGCTC | 204 bp | 8849098 |
| InDel-50-R | AAAATGACTCGTCCGATAT | | |
| InDel-55-F | ATTTTCTTTTGGTCGTTGC | 232 bp | 9331829 |
| InDel-55-R | CCTTCGCTAGTTAATGATATTTG | | |
| InDel-56-F | CCGTATCCATCGGAGCAGA | 274 bp | 9341540 |
| InDel-56-R | CGTTCGCCGTGAGGAGTGT | | |
| InDel-57-F | CATACAATACCTTCAACACCCT | 266 bp | 9360843 |
| InDel-57-R | TTTGGCTGTTTCAGGTCAG | | |
| InDel-65-F | AAGCAGTGATGAAGACGA | 226 bp | 9517527 |
| InDel-65-R | TCTCATTGCCAAAGATTAT | | |
| InDel-67-F | TCCCATCTTCCCACCTGA | 248 bp | 9643657 |
| InDel-67-R | TCCTTCCTAAGCGTGACTTG | | |
| InDel-70-F | TCCAAGGAGGAAGAAGCG | 245 bp | 10090197 |
| InDel-70-R | CCAATAACTACCAATAACATCTAAAA | | |
| InDel-72-F | TCTCAAATGGAAGGGAAGT | 239 bp | 10091868 |
| InDel-72-R | TTGTCGGCCACATTACTC | | |
| InDel-73-F | CACAACATGCCACTAAACC | 180 bp | 10108987 |

| | | | |
|---|---|---|---|
| InDel-73-R | AAGCAGAACAACCGAAGA | | |
| InDel-75-F | TGGATTTTAGGGTAGTTTT | 224 bp | 9968729 |
| InDel-75-R | AGGGCATCATCAGGTTCT | | |
| InDel-78-F | AAACACTCAAGGGCTTAG | 239 bp | 10055390 |
| InDel-78-R | TTTTAACGTCGTCAGAAA | | |
| InDel-80-F | GCTCCAGTGGTTGTTCTGT | 202 bp | 10071605 |
| InDel-80-R | ATCGTCTTTCGTCGTTCATA | | |
| InDel-81-F | CACATCAAATTAAACAAGAAT | 193 bp | 10077900 |
| InDel-81-R | GAAACCATCATCATCAACA | | |
| ID-62-F | GTGCCTTTAATAGCCTCTG | 243 bp | 36995890 |
| ID-62-R | CTTTCTGCTGTAGACCAACT | | |
| ID-63-F | TTTCTTGGTATGTATCGCCC | 244 bp | 36997514 |
| ID-63-R | CAGGTAACCACTATTCCATTTT | | |
| ID-64-F | AAACAAACCATTCTTCACA | 251 bp | 37002642 |
| ID-64-R | TTGATTTGGATCTATGTGC | | |
| ID-65-F | CGATTTTATTGCTCCTTTG | 232 bp | 37003529 |
| ID-65-R | TTTTCAATTCCAGGTTTAGAG | | |
| ID-66-F | CGCAGGCACTAATAAATGA | 220 bp | 37006486 |
| ID-66-R | TATGCAAAACGAAAAGGAA | | |
| ID-67-F | ACCCAACCCAAAACTAAAG | 202 bp | 37014528 |
| ID-67-R | GTGGTTTGTGAAGTATTATGTGA | | |
| ID-68-F | AAGATCAGGCCAACGAATT | 248 bp | 37148339 |
| ID-68-R | CCACCAGAGTAACGAGCATAT | | |
| ID-70-F | TTCAGTGGTTAAAGTCAATG | 227 bp | 37583089 |
| ID-70-R | TACGATTTATTCAACCAACT | | |
| ID-73-F | ATTCCCTATGTCTATTCGT | 254 bp | 37633145 |
| ID-73-R | CTGTCTTACGCTAGAGTTT | | |
| ID-74-F | CCGTTAAGTGAGGGTTCAT | 271 bp | 37669709 |
| ID-74-R | AAGATTCTGTCCGGTTGTC | | |
| ID-77-F | TAAAGTAGCTGCCGGTCTG | 221 bp | 37740130 |
| ID-77-R | GAACATTACGGTAAGGGTGAG | | |
| ID-78-F | ACCCTAACCATCCTTCTAATGC | 227 bp | 37770123 |
| ID-78-R | CGTCGGAACAAGAGCTAACC | | |
| ID-79-F | TTATTTCAGGTGGCTACGG | 217 bp | 38049836 |
| ID-79-R | TGTCCTTGTTATTCGGGTT | | |
| ID-81-F | TTCAACTGCCACAAGATTT | 238 bp | 38251729 |
| ID-81-R | AGAGGTTTGTCTTTTGCTAC | | |
| ID-82-F | ATAAAAGGACCAGACAATA | 249 bp | 38278750 |
| ID-82-R | TGAACACGCTAGTAGAAGT | | |
| BnA05ID1-F | ACCATACAACCGTGTCAAAC | 120 bp | 10083368 |
| BnA05ID1-R | GAGCAGCGAAACACCAG | | |

**Table S4.** The designed primers of DNA sequencing used in this study.

| Name of Primers | Sequence of Primers | Product Length |
|---|---|---|
| BnaA05G0157300ZS-F | GATAGAAGGAGTAGAAACATTAG | 291 bp |
| BnaA05G0157300ZS-R | GAGAGAAAAGGGTAGAACAAG | |
| BnaA05G0157400ZS-F | ATGCTTCTTATCTTCCT | 918 bp |
| BnaA05G0157400ZS-R | TGAATCAAATACCTGTC | |
| BnaA05G0157500ZS-F | AAGTGACGATTGGCGAAGA | 882 bp |
| BnaA05G0157500ZS-R | CAGATTTATTTAGAGTTTGGACA | |
| BnaA05G0157600ZS-F | GTGTGTAGCAAATGTGAT | 509 bp |
| BnaA05G0157600ZS-R | GTCACTTGCTAAATCCC | |
| BnaA05G0157700ZS-F | ACAATAGAACTGCTACATTAGATGC | 2184 bp |
| BnaA05G0157700ZS-R | CATAAATATTGATAATCCAACACAC | |
| BnaA05G0157800ZS-F | GTCTATCTTCTGGGTTTACTTA | 1550 bp |
| BnaA05G0157800ZS-R | CGAAGTAGACGCAAAGC | |
| BnaA05G0157900ZS-F | CAAAAACCTTACCAGCATCC | 1539 bp |
| BnaA05G0157900ZS-R | ATAAACTTTGTAATAAGAACCCAGA | |
| BnaA05G0158000ZS-F | AAAATATCAAAGACTTAGCGAG | 2402 bp |
| BnaA05G0158000ZS-R | CCTCTTCCACCATCTTCTA | |
| BnaA05G0158100ZS-F | ATACATGTCACACTTCTCGTAAAAC | 1103 bp |
| BnaA05G0158100ZS-R | CTTGATGTGATAATCTTTCGTGT | |
| BnaA05G0158200ZS-F | GCCTGAGAAGTTAAGCATG | 501 bp |
| BnaA05G0158200ZS-R | AACCCTACAGGCACTTGA | |
| BnaA05G0158300ZS-F | TGGAGAATTGTTTACTAACCTG | 2698 bp |
| BnaA05G0158300ZS-R | TCTGTGGCTTGTCCTTCTTA | |

**Table S5.** The designed primers of CDS sequencing used in this study.

| **Name of Primers** | **Sequence of Primers** | **Product Length** |
|---|---|---|
| BnaA05G0157300ZS-CDS-F | ATGGCAAAATGGAGTGCAATAG | 234bp |
| BnaA05G0157300ZS-CDS-R | TTAGTACCTAGGGGGAGGAGG | |
| BnaA05G0157400ZS-CDS-F | ATGCTCGCTGATTTGGAGGCCG | 360 bp |
| BnaA05G0157400ZS-CDS-R | TCATAACTGGGAGCAGCTGGAG | |
| BnaA05G0157500ZS-CDS-F | ATGGTTAATATGTCTCAGTCTC | 162 bp |
| BnaA05G0157500ZS-CDS-R | TTACTTCATTGCTAGTTTTAC | |
| BnaA05G0157600ZS-CDS-F | ATGAAGAGAGTATCTGCAGAGC | 225 bp |
| BnaA05G0157600ZS-CDS-R | TTACAAGATAGAAGAAGAAGG | |
| BnaA05G0157700ZS-CDS-F | ATGGACATCACAAGAAACAGC | 1260 bp |
| BnaA05G0157700ZS-CDS-R | TTAGTGACAAGTATTAGTGCAAGG | |
| BnaA05G0157800ZS-CDS-F | ATGGTGATTTGTGATGTTGTTG | 735 bp |
| BnaA05G0157800ZS-CDS-R | TCAAAGACGACTGATGACTGC | |
| BnaA05G0157900ZS-CDS-F | ATGGATTCAGTGACATCCTTC | 1188 bp |
| BnaA05G0157900ZS-CDS-R | CTATAAACCGGAGCCTGATCG | |
| BnaA05G0158000ZS-CDS-F | ATGGCACCAATAAAAGATCTC | 1758 bp |
| BnaA05G0158000ZS-CDS-R | TCAAAGACTTAGCGAGAAAGGG | |
| BnaA05G0158100ZS-CDS-F | ATGGACGAAGAATCTGCTTC | 948 bp |
| BnaA05G0158100ZS-CDS-R | TCAAAGATTGCTGTTATTGG | |
| BnaA05G0158200ZS-CDS-F | ATGGCAGACGATTTAAATACTG | 318 bp |
| BnaA05G0158200ZS-CDS-R | CTAAAAATCTTTGTTCTTCCG | |
| BnaA05G0158300ZS-CDS-F | ATGACTCTTTCAGATATTGCTAC | 1440 bp |
| BnaA05G0158300ZS-CDS-R | TTAGACTCTGGTTCCGTTTGGC | |
| BnaA05G0157900UD-CDS-F | ATGGATTCAGTGACATCCTTC | 1761 bp |
| BnaA05G0157900UD-CDS-R | TCAAGATAGCTGATGGGTCACTC | |
| BnaA05G0158100UD-CDS-F | ATGGACGAAGAATCTGCTTC | 951 bp |
| BnaA05G0158100UD-CDS-R | TCAAAGATTGCTGTTATTGG | |

**Table S6.** The designed primers of quantitative RT-PCR used in this study.

| Name of Primers | Sequence of Primers | Product Length |
|---|---|---|
| BnaA05G0157900ZSRT-F | GATCACGAAGCAGCTCAAATC | 248 bp |
| BnaA05G0157900ZSRT-R | CTTCGTTCACCGTAGTCACATC | |
| BnaA05G0158100ZSRT-F | CGAAGAATCTGCTTCTAATGGC | 134 bp |
| BnaA05G0158100ZSRT-R | TAGCGTAGAGATGAAGCAAGAG | |
| BnaA05G0158300ZSRT-F | CAGACGAACAAGAGTTTCTGTG | 130 bp |
| BnaA05G0158300ZSRT-R | GATTTTGGAGCTCTTCTGTTGG | |
| BnActin-RT-F | ATTCAGCCCCTTGTTTGTG | 147 bp |
| BnActin-RT-F | GTAAGCGTCTTTTTGACCCAT | |
